# Supplementary material for: Structure and Dynamics of Compact Dinucleosomes: Analysis by Electron Microscopy and spFRET
Source: Int J Mol Sci. 2023 Jul 28;24(15):12127. doi: 10.3390/ijms241512127 (PMC10419094; doi:10.3390/ijms241512127)
Supplement: Supplementary file 1 [file ijms-24-12127-s001.zip › ijms-2430416-supplementary.pdf]

Structure and Dynamics of Compact Dinucleosomes: Analysis by Electron Microscopy and spFRET

Maria E. Stefanova, Olesya I. Volokh, Oleg V. Chertkov, Grigory A. Armeev, Alexey K. Shaytan, Alexey V. Feofanov, Mikhail P. Kirpichnikov, Olga S. Sokolova, Vasily M. Studitsky

SUPPLEMENTARY MATERIALS

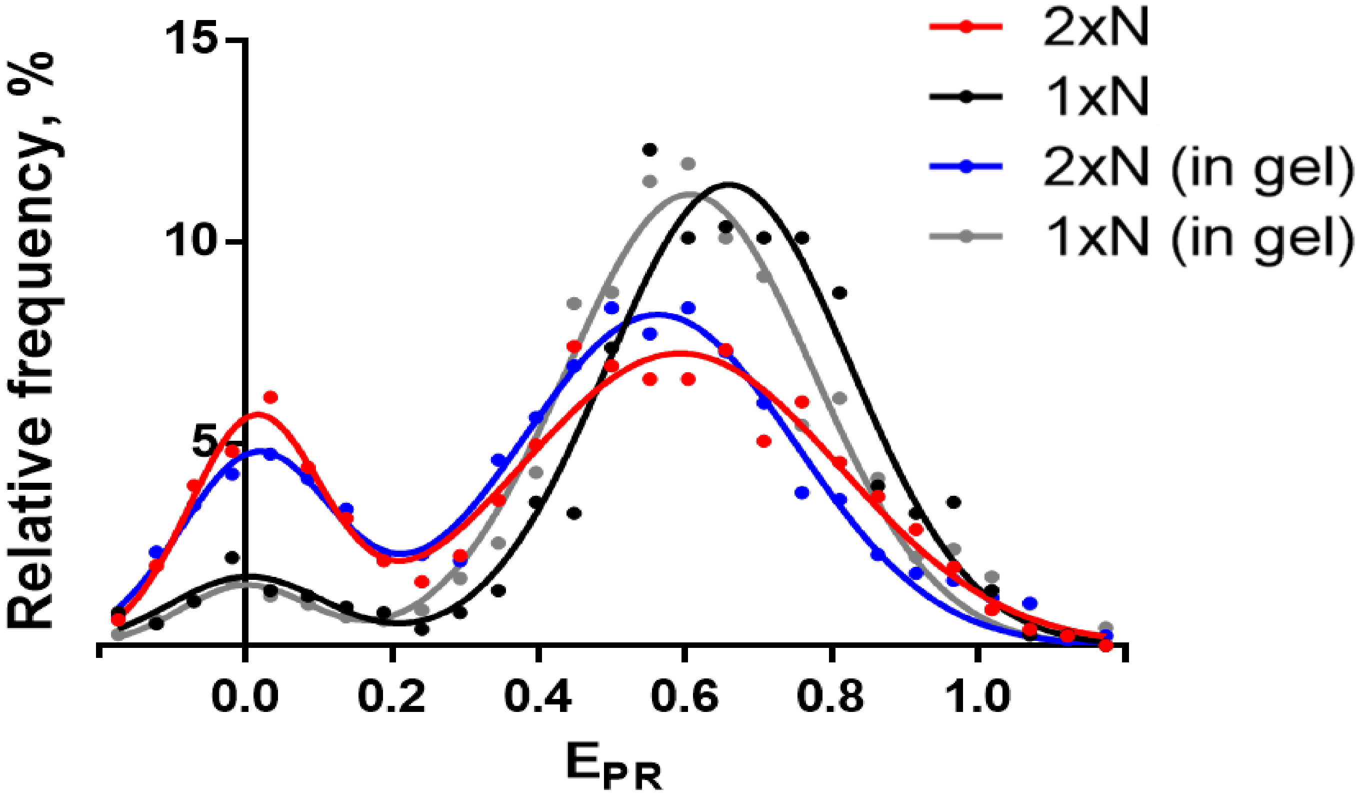

Figure S1. Analysis of mono- (1×N) and dinucleosomes (2×N) in solution and in gel using spFRET approach. Nucleosomes were labeled at positions 13/91 bp. Designations as in Figure 2b.

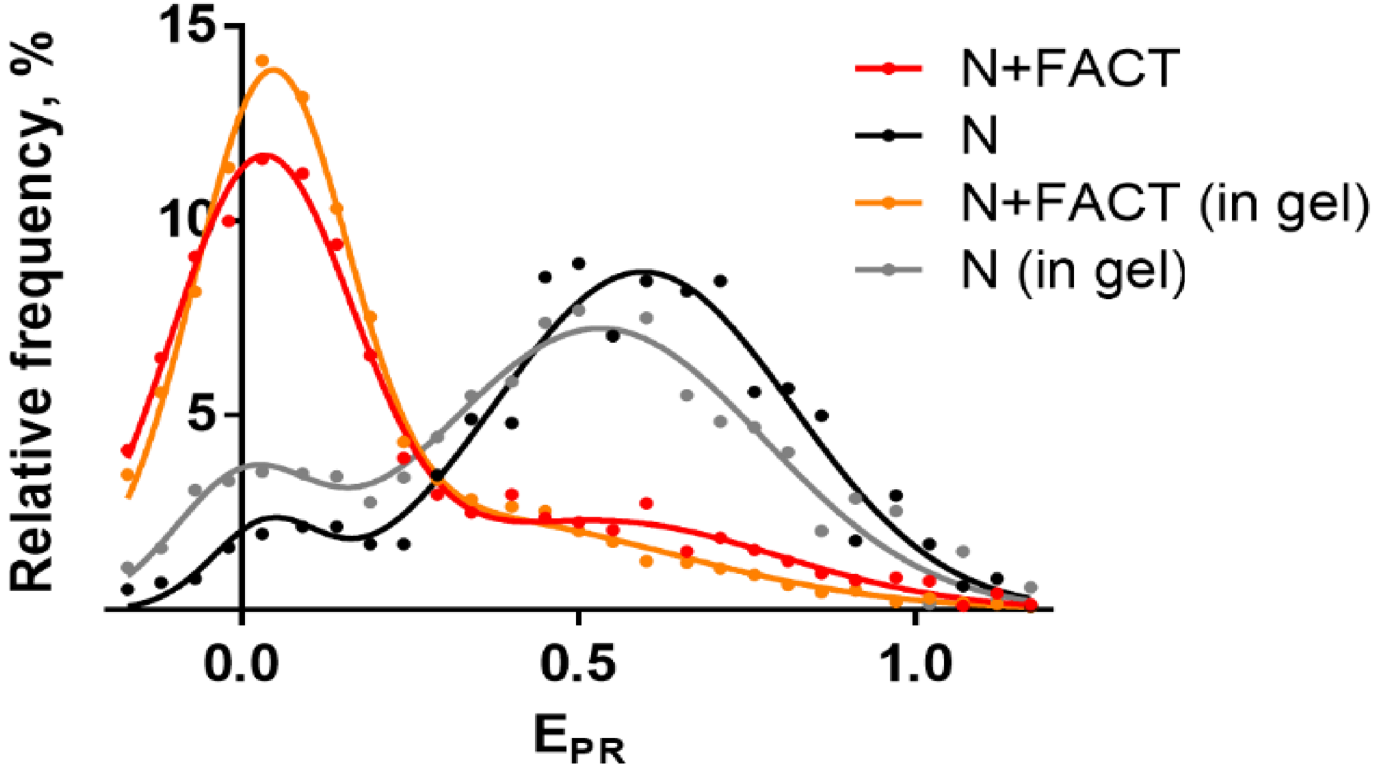

Figure S2. Analysis of nucleosome unfolding by yFACT in solution and in gel using spFRET approach. Nucleosomes were incubated in presence/absence of yeast FACT. Nucleosomes were labeled at positions 35/112 bp. Designations as in Figure 2b.

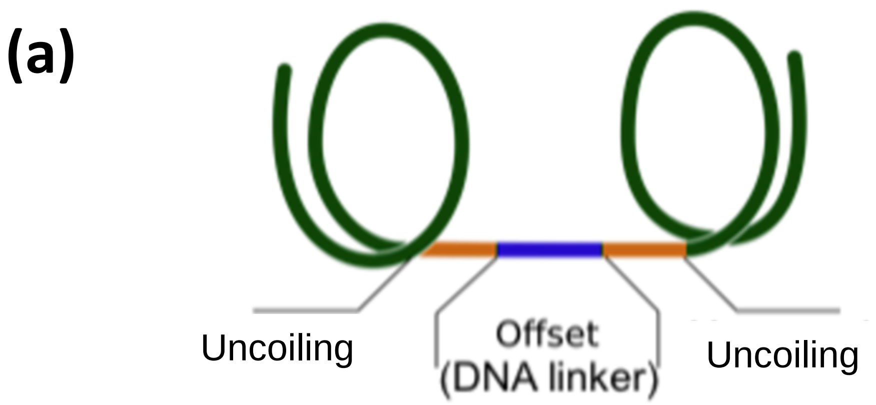

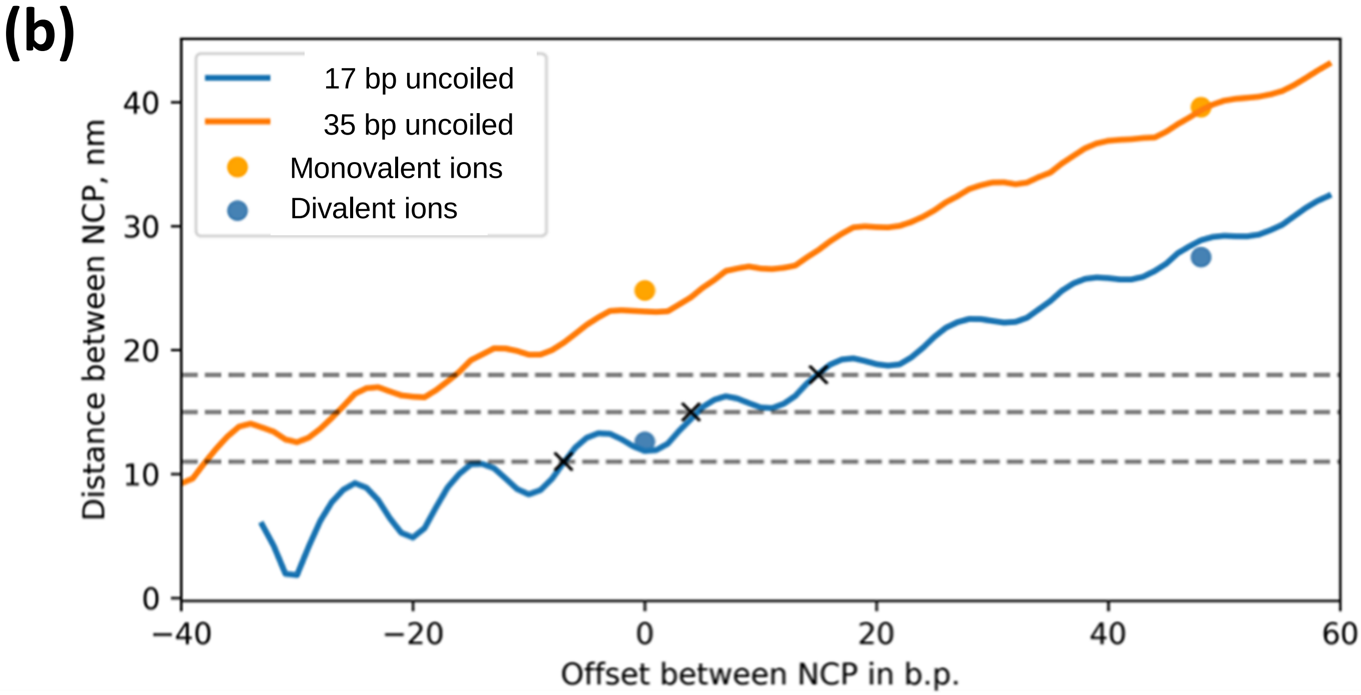

Figure S3. Dependence of the inter-nucleosome distances on the extent of uncoiling of nucleosomal DNA from the histone octamer. (**a**) Schematic representation of the model of the dinucleosome system. (**b**) Dependence of the inter-nucleosome distance on the offset between nucleosomes for different cumulative uncoiling values. Blue and orange lines represent theoretical profiles of the dependence of the inter-nucleosome distance on the offset between nucleosomes for two different DNA uncoiling values (17 bp and 35 bp, respectively). Blue and orange circles depict experimental data points; the distance between nucleosomes was measured using atomic force microscopy (10). Dashed lines correspond to the distances between nucleosomes observed in the three main classes identified by our electron microscopy studies. The intersections between the dashed lines and the theoretical profile are marked by crosses.

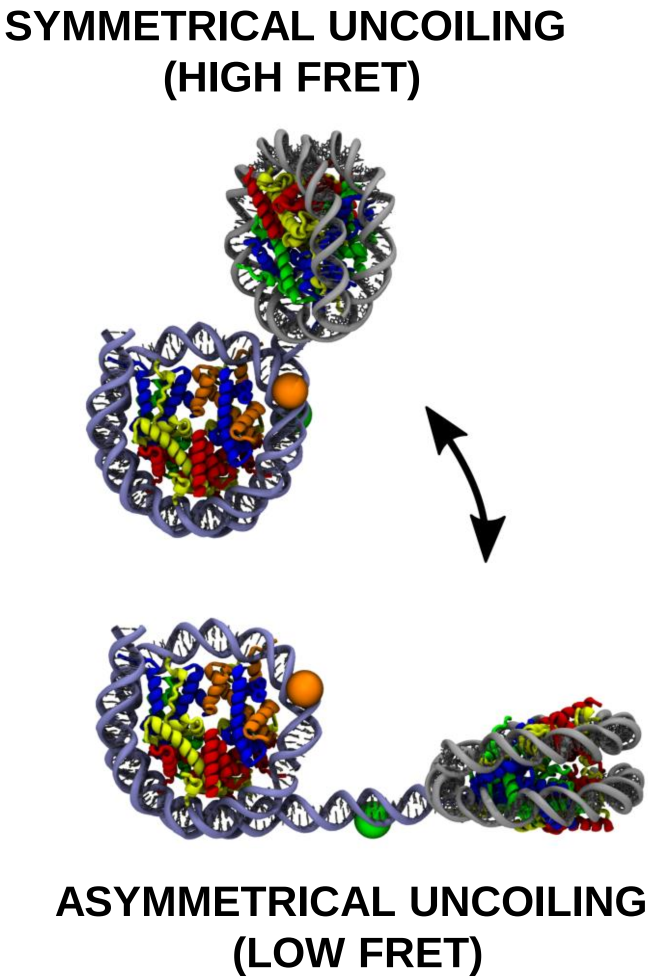

Figure S4. Examples of models with the same offset and different DNA uncoiling modes: symmetrical (top) and asymmetrical uncoiling (bottom). High FRET efficiency is expected for the model with symmetrical DNA uncoiling. Low FRET efficiency is expected only for the model with asymmetrical DNA uncoiling. Labels are shown with colored circles.

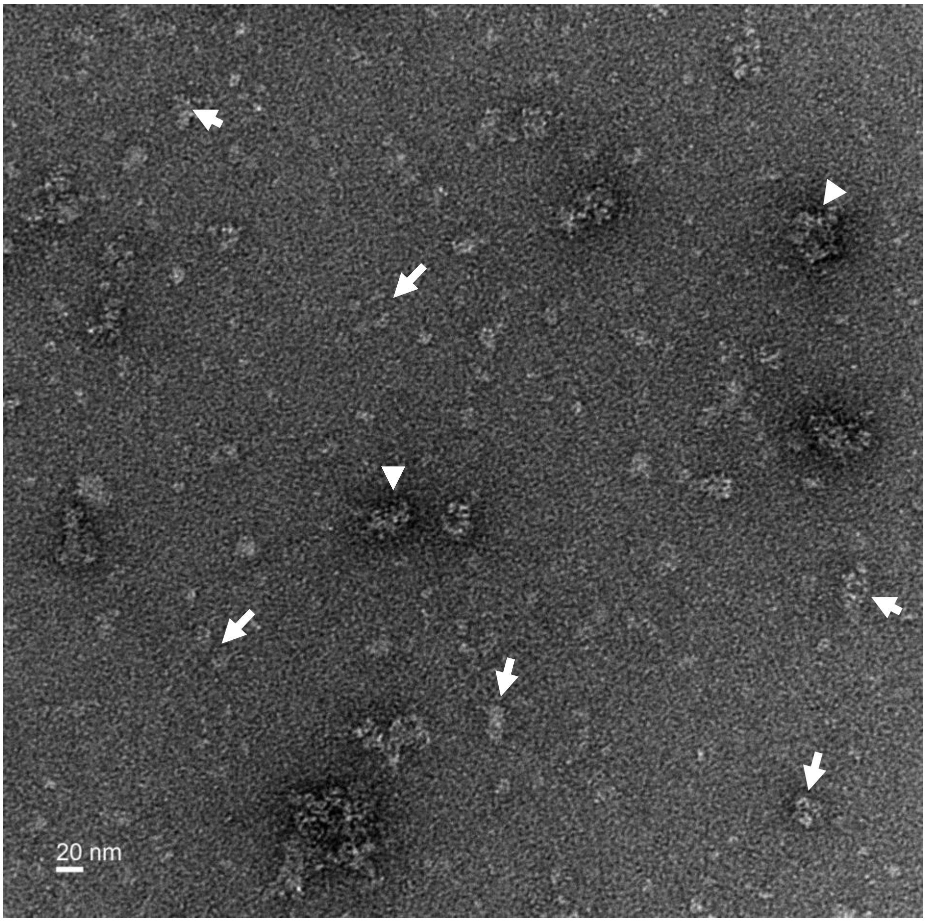

Figure S5. Raw image of negative stained dinucleosomes sample. Arrows indicate dinucleosomes; arrowheads – aggregates. There are ~25% of single particles in the dinucleosome preparation that are similar in size to mononucleosomes. The presence of these particles could be explained by particular orientation of the dinucleosomes on the grid, where one particles is not visible. However, we cannot exclude a possibility that the presence of these particles is explained by the presence of contaminations (e.g. BSA or particles of the gel used for preparation of the samples).

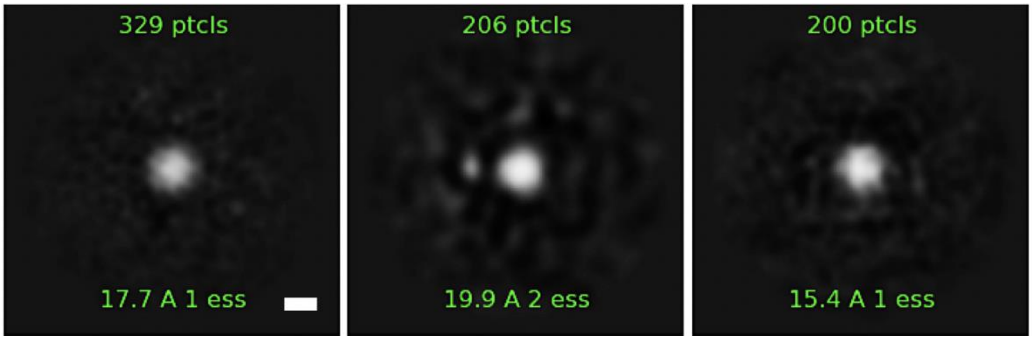

Figure S6. Typical class-averages of mononucleosome-size single particles present in the dinucleosome preparation. ~25% of single particles in the dinucleosome preparation have this appearance. Scale bar 10 nm.
